# Clocks, events, and the ontology of perceiving and acting

**DOI:** 10.3389/fpsyg.2026.1858938

**Published:** 2026-07-28

**Authors:** Thomas A. Stoffregen, Robert Heath

**Affiliations:** 1 Virtual Reality Laboratory, University of Minnesota, Minneapolis, MN, United States; 2 Independent Researcher, Winona, MN, United States

**Keywords:** ecological psychology, event perception, metrology, ontology, perception, time

## Abstract

James Gibson introduced the concept of ecological physics. Gibson asserted that contemporary physics, such as general relativity, was not relevant to perceiving and acting. One area in which Gibson claimed that ecological physics differed from contemporary physics was in concepts of time and temporality. Gibson argued that time is intrinsic to physical events that are spatially and temporally extensive, and is not (as in general relativity) an abstract medium within which events occur. In this article, we contrast Gibson’s concept of time with time as it is conceptualized within general relativity, in which time is extrinsic to physical events. We do not criticize general relativity as a theory of physics. Rather, we argue that Gibson’s concept of intrinsic time in ecological physics is not compatible with concepts of time used in general relativity. We develop our argument through an analysis of clocks. We argue that clocks do not detect the abstract, extrinsic concept of time of general relativity but, instead, detect only physical events that are extensive in time and space. For that reason, we suggest that clocks conform with ecological physics, rather than with general relativity. We further argue that the concept of extrinsic time that is central to general relativity should not be taken as the default in behavioral science studies of “time perception.” In conclusion, we argue that Gibson’s concept of time as being intrinsic to physical events has implications for our understanding of how perceiving and acting in the behavioral sciences relate to concepts in general relativity.

## 1 Introduction

The Ecological Approach to Perceiving and Acting is an established theory of behavior that has been widely influential in understanding animal-environment interactions, such as manual manipulation (e.g., Ranganathan et al., 2011), locomotion (Warren et al., 2001), development (e.g., Adolph, 2020), and interpersonal and social interactions (e.g., Richardson et al., 2007). The ecological approach is understood to have implications in technological settings, such as driving (e.g., Fajen, 2005), and interface design (e.g., Mesgari et al., 2023), and in clinical interventions in physical rehabilitation (e.g., Varoqui et al., 2011) and mental health (e.g., Gadsby and Williams, 2018), as well as for philosophy (e.g., Sanders, 1999). The ecological approach has its origins in the work of Gibson (Gibson, 1966, 1979). Gibson offered arguments about the ontology of perceiving and acting, and about the epistemology of perceiving and acting. In terms of ontology, Gibson, 1979 claimed that perceiving does not provide information about all of physical reality. Rather, Gibson claimed that perceiving provides information about physical events at the scale of perceiving and acting, and about affordances, that is, opportunities for action that emerge from relations between properties of the animal and properties of the environment, which can include other animals (e.g., Stoffregen, 2003). In terms of epistemology, Gibson claimed that events and affordances, as parts of the physical ontology, impart structure to ambient energy arrays, such as light, sound, or the global array (Stoffregen et al., 2017), and that the structuring of such arrays is governed by physical law. Therefore, the patterns in ambient arrays bear a unique, 1:1 relation to those events and affordances, such that those events and affordances are specified in patterns that are ambient to the animal and which may be sampled by perceptual systems (e.g., Gibson, 1979; Stoffregen and Bardy, 2001; Turvey, 2018). On this basis, Gibson claimed that patterns in ambient energy arrays are sufficient for accurate perception of events and affordances, such that perceiving can be direct, that is, accurate without the need for any internal, inferential processing of ambient information.

In this article, we examine Gibson’s arguments about time in the context of one of the most ubiquitous instantiations of concepts of temporality–the use of clocks in the measurement of time. We discuss clocks in relation to the ontology of time, asking what it is that clocks actually detect. We also discuss clocks in relation to the epistemology of time, asking about what it is that clocks detect in relation to the perceiving of temporality by living things. The interplay between ontology and epistemology is important for three reasons. First the ontology determines what is available to be perceived; that is, we should expect to perceive only things that are part of the physical ontology. Second, the nature of the ontology may have critical implications for how perceiving works or, in a Darwinian system, how it should work (in the sense of being most adaptive): As an activity, perceiving should be adapted to be sensitive to aspects of the physical ontology. Finally, analysis of what actually is (and is not) perceived can inform our understanding of the nature of perceptual ontology: Things that are perceived should be part of the physical ontology, while things that are not perceived may not be part of the physical ontology. While the focus of our analysis is on time, the logic of our arguments applies equally to the ontology and epistemology of space. In Section “4 Time in information processing,” we briefly discuss this issue.

We contrast Gibson’s arguments about time with time as it is understood by physicists in general relativity. We focus on events, rather than on affordances, because events play important roles in both general relativity and in the ecological approach to perceiving and acting, whereas the concept of affordances is not found in general relativity. We do not criticize general relativity as a theory of physics. Rather, we make two claims. First, we claim that Gibson’s concept of time as being intrinsic to events is distinct from and incompatible with general relativity. Second, we claim that Gibson’s concept of intrinsic time should be the default for the behavioral sciences. That is, we claim that the perceiving of time and the control of time-dependent action is best understood in terms of Gibson’s concept of time being intrinsic to events, without reference to or dependence upon concepts of time used in general relativity.

### 1.1 Definitions

In this section, we define terms that are central to our discussion, and which have technical definitions that are critical to distinguishing our arguments from those of other authors.

The concept of events is critical to our analysis. This term is used in both general relativity and by Gibson, but in very different ways. To avoid confusion, we distinguish between the definitions of event offered by general relativity and Gibson. In addition, we offer a third definition that is unique to our discussion. The three definitions are distinguished by subscript labels:

Events_*GR*_: The definition of event used in general relativity: “…an instantaneous occurrence that can be characterized by a point in space and a corresponding moment of time,” (Fock, 1964, p. 33), where “point in space” is understood to be a 1-dimensional geometric point having no spatial extent, and “moment of time” is understood to be instantaneous, having no temporal extent (i.e., no duration). Similarly, in general relativity event time refers to the exact temporal coordinate or instant assigned to an event by an observer in a specific reference frame.

Events_*E*_: Gibson discussed and described ecological events. He did not offer a formal or final definition (e.g., Gibson, 1979, p. 94), but he provided enough detail to make clear that his concept of event differs qualitatively from Events_*GR*_. For Gibson, events have duration and occupy space, that is, they are not instantaneous and do not occur at geometric points. Gibson typically referred to events as existing at the ecological scale, that is, at the scale of perceiving and acting (Gibson, 1979, p. 8–12). Thus, a ball rolling down a hill would constitute an Event_*E*_, but the splitting of an atom would not. The category of Events_*E*_ differs across species and individuals, depending upon their perceptual sensitivity. For example, events that structure ultrasound are Events_*E*_ for bats, but not for humans, while events that structure infrasound are Events_*E*_ for whales but not for humans.

Events_*P*_: Physical events. Our definition is the same as Gibson’s definition of Events_*E*_ except that it is not limited to events at the ecological scale. For us, Events_*P*_ are any physical events, regardless of scale, that have duration and occupy space. Thus, Events_*P*_ is a superordinate category that contains Events_*E*_, such that all Events_*E*_ are Events_*P*_, but some Events_*P*_ are not Events_*E*_. At the same time, Events_*P*_ are distinct from the timeless, spaceless events of general relativity (Events_*GR*_).

Local time, or Coordinate time: In general relativity, the time measured by a grid of synchronized clocks scattered throughout a stationary observer’s reference frame. Coordinate time is defined in relation to the observer’s relative speed and their gravitational environment. Because of time dilation, different observers will assign different coordinate times to the same Events_*GR*_.

Proper time, or Clock time: In general relativity, the time measured by a clock that is moving with the object being observed. It is the time recorded on a single, local clock that is present at both the start and end of an Event_*GR*_. Because it is a physical measurement of a journey through space-time, all observers in all frames of reference will agree on exactly how much proper time elapsed for that specific object.

Extrinsic time: Time that exists (and passes) independent of Events_*GR*_, Events_*P*_, and Events_*E*_. Extrinsic time is understood as a “container” for Events_*GR*_ and Events_*P*_, that is, as a framework within which Events_*GR*_, Events_*P*_, and Events_*E*_ transpire.

Intrinsic time: Time that is intrinsic to Events_*P*_ (including Events_*E*_), such that the passage of time occurs only with (or as a property of) Events_*P*_. Time as a property of Events_*P*_ is not defined with reference to any external (i.e., extrinsic) frame of reference.

Perception: The pickup of information or the activity of picking up information by living things. Living things perceive, whereas non-living things (such as clocks) may detect but do not perceive. Perception can lead to conscious awareness, but awareness is an outcome of perception, rather than being a defining part of the activity.

Detection: Physical sensitivity to the occurrence of events. A thermometer used to measure temperature may be based upon detection of different physical facts, such as expansion, because heat causes things to expand, electrical resistance, because heat causes changes in electrical resistance of materials, or infrared radiation, because greater heat leads to greater infrared radiation (e.g., Chang, 2004). Likewise, clocks used to measure time may be based upon detection of a wide variety of physical facts.

Measurement: Interpretation of data in terms of theoretically defined quantities (e.g., Mari, 2003). Thus, while a thermometer may detect (for example) infrared radiation, it can be said to measure temperature when the detected radiation is scaled to an accepted standard. In science, in general, and behavioral science, in particular, measurement emerges from a network of theoretical commitments, instruments (such as clocks), and standards (Michell, 1997). Theoretical commitments and standards are types of social conventions, in the senses that (1) they are agreed upon by people, and (2) their values are subject to change with changes in those human agreements. Clocks and other instruments are physical devices, but the choice of which instruments to use in measurement is subject to the same theoretical commitments and standards. The dependence of instruments on social conventions is illustrated by the fact that instruments that are accepted as standard at one point in time (e.g., electric clocks) are no longer accepted at a later time (e.g., after it was agreed that the atomic clock would be the standard for time-keeping).

### 1.2 Gibson’s intrinsic time

The ecological approach to perceiving and acting diverges from traditional theories of behavior in many ways. These include claims about ontology (i.e., the nature of things that might be perceived) and epistemology (i.e., how perceiving works). In terms of ontology, theories of perceiving traditionally have accepted time as it has been defined in physics, where time is an extrinsic dimension, such that time is absolute in the sense of being independent of Events_*P*_ (including Events_*E*_). Such theories predict that this “classical time” is perceived, and attempt to explain our awareness of the passage of time in terms of clock-like neural mechanisms (e.g., Ivry and Schlerf, 2008). In developing the ecological approach to perceiving and acting, Gibson (1966, 1975, 1979) made novel claims about the ontology of time, and about the epistemology of time. In terms of ontology, Gibson claimed that time does not exist as an absolute, extrinsic temporal framework. Gibson claimed that, rather than being extrinsic, time was an intrinsic property of Events_*P*_. In terms of epistemology, Gibson claimed that the temporal structure of Events_*E*_ can be perceived (e.g., a ball rolling downhill), but that extrinsic time as construed in general relativity cannot be perceived (e.g., the passage of seconds, or minutes). As part of the broader claim that perception can be direct, Gibson argued that the temporal structure of Events_*E*_ lawfully structures ambient energy arrays, such that animals sensitive to such arrays might directly perceive the temporal structure of Events_*E*_. By contrast, Gibson argued that time as it is understood in general relativity does not lawfully structure ambient energy arrays and, therefore, it cannot, in principle, be perceived directly.

In the definition that is operationalized in clocks, time is understood to pass with or without the occurrence of Events_*P*_ (e.g., Tal, 2016; Thomas et al., 2026). The extrinsic nature of temporal units means that such units (e.g., seconds) are uniform across all situations, locations, and circumstances (Audoin and Guinot, 2001). For processes that involve long distances and extremely precise temporal synchronization, temporal effects of special and general relativity must be taken into account; a common example is found in satellite navigation systems such as the Global Positioning System (e.g., Ashby, 2003). However, for most terrestrial scientific applications, and universally in daily life, clocks are understood to give us access to an abstract, absolute sequence of discretely bounded, identical temporal units, such that 1 s (for example) in Sydney is identical to 1 s in Nome, 1 s in January is identical to 1 s in June, and 1 s in the morning is identical to 1 s at night. As noted by Audoin and Guinot (2001, p. 36), “in everyday life, ordinary Newtonian time is quite sufficient for most technical and scientific applications.”

### 1.3 Intrinsic time in general relativity?

In certain senses, under general relativity time is relative. For example, the Events_*P*_ that are detected by clocks differ for clocks moving at different velocities, suggesting that time varies across locations and situations. This fact might seem to undermine our characterization of time in general relativity as being absolute. However, in general relativity those aspects of local time (i.e., proper time) are, themselves understood to be embedded within a larger framework that is absolute (e.g., Ashby, 2003; Audoin and Guinot, 2001; Petit et al., 2014). This is so because the comparison of clocks that are moving at different velocities can be accomplished only with respect to the speed of light. In general relativity, the speed of light is absolute and constant. That is, the speed of light has the same observed value from all points of observation, regardless of observer velocity. Thus, for each locality (i.e., each velocity), local time is defined relative to the (absolute) speed of light. These are examples of the overarching fact that the entire mathematics of general relativity depends upon the absolute invariance of the speed of light, which serves as the essential conversion factor linking spatial and temporal dimensions into a unified geometric framework. Without the assumption of an invariant speed of light that is extrinsic to Events_*GR*_, the fundamental equations of general relativity are not internally consistent (e.g., Guidry and Guidry, 2019). For these reasons, we claim that the concept of time in general relativity differs fundamentally from Gibson’s concept of intrinsic time. For Gibson, time is intrinsic without reference to the speed of light or any other extrinsic frame of reference. In the remainder of this section, we present this point in relation to several aspects of general relativity.

An important empirical prediction derived from general relativity is that proper time should differ between clocks traveling at different velocities. The advent of space travel has made it possible to test that prediction. Data collected from spacecraft have been consistent with predictions derived from general relativity (e.g., Petit et al., 2014; Qin et al., 2019). It might be argued that such effects are evidence that time can be relative or intrinsic, rather than absolute or extrinsic. We accept the empirical results, but we reject the claim that they are consistent with Gibson’s concept of intrinsic time. In relativistic clock comparisons, the velocity of the clocks can be defined only relative to the speed of light, such that the comparison itself assumes an extrinsic framework. Gibson’s concept of intrinsic time rejects a role for any extrinsic framework and, thus, is not compatible with the data on relativistic clock comparisons.

In general relativity, clocks act as tools that measure paths through the four-dimensional fabric of spacetime. Rather than ticking at a constant universal rate, clocks tick at rates dictated by their velocity and the local gravitational field, directly mapping out the geometric structure of spacetime itself. It might be argued that such effects are evidence that time can be relative or intrinsic, rather than absolute or extrinsic. We accept the conceptual role of clocks in understanding the geometry of the general relativity model, but we argue that such a role is inconsistent with Gibson’s concept of intrinsic time. The notional role of clocks in mapping the geometry of spacetime requires the assumption that the velocity of the clocks can be defined only relative to the speed of light. Thus, as with the empirical data on relativistic clock comparisons, the conceptual role of clocks in mapping the geometry of spacetime assumes an extrinsic framework – the extrinsic speed of light. As noted above, Gibson’s concept of intrinsic time rejects a role for any extrinsic framework and, thus, is not compatible with the conceptual role of clocks in mapping the structure of spacetime.

As noted in Section “1.1 Definitions,” proper time, or clock time along a timelike world line is defined as the time measured by a clock following that line. In general relativity, clock time differs for clocks on different world lines, which might seem to be a clear example of time being intrinsic to different Events_*GR*_ (in this case, different world lines). Yet, we claim, clock time (as defined by general relativity) is not compatible with Gibson’s concept of intrinsic time. In general relativity, all quantities ultimately must be tied to the speed of light which, by definition, is extrinsic and absolute. Thus, despite its local characteristics, clock time differs qualitatively from Gibson’s concept of time that is intrinsic to Events_*P*_.

As noted in Section “1.1 Definitions,” coordinate time, or local time is the time indicated by a grid of synchronized clocks within an observer’s reference frame. Because of time dilation, different observers will assign different coordinate times to the same Events_*GR*_, which might suggest that local time is intrinsic to particular reference frames. We argue that local time is not compatible with Gibson’s concept of intrinsic time, because the clocks in the grid are synchronized only relative to the speed of light, which is extrinsic. We conclude that local time differs qualitatively from Gibson’s intrinsic time.

Under general relativity, the speed of light – construed as being fixed, absolute, and extrinsic to Events_*GR*_, is the fundamental conversion factor that makes it possible for the mathematics of the theory to achieve internal consistency. In this section, we have argued that any concept of time that emerges from or is consistent with general relativity cannot be consistent with Gibson’s concept of intrinsic time, because the latter eschews extrinsic frameworks of any kind. In the next section, we consider the physical function of clocks (and their evolution as a technology) in relation to concepts of time and the detection and measurement of time.

## 2 Measuring time through history

Because measured time is a central concept in general relativity, clocks have always played a critical role in both the conceptualization and the empirical evaluation of the theory. In this section, we review the technological history of clocks (cf. Levine, 1997). We note that each technology that has been used in the measurement of time is sensitive to Events_*P*_, and is not sensitive to the hypothetical extrinsic time that is claimed to pass independent of Events_*P*_. On this basis, we reject the commonplace idea that clocks “measure time,” or even detect time. We point out that the interpretation of detected Events_*P*_ in terms of extra-physical, extrinsic time is just that – an interpretation, and not a fact. As an alternative interpretation, we suggest that clocks’ sensitivity to Events_*P*_, combined with their insensitivity to extrinsic time is akin to Gibson’s (1966, 1975) claim that living things perceive Events_*E*_ but cannot perceive extrinsic time. The different technologies are presented in chronological order. Our historical review illustrates the consistent use of technologies that detect Events_*P*_, and sets up our later arguments relating to Gibson’s claim that living things can perceive Events_*E*_ but cannot perceive time.

The technological evolution of horology has tended toward improvements in precision, accuracy, and synchronization (cf., Audoin and Guinot, 2001). By precision, we refer to the duration of the Events_*P*_ that are detected. Improvements in precision enable the reliable detection of phenomena of decreasing duration, from hours to minutes to seconds to smaller and smaller fractions of seconds. By accuracy, we refer to the consistency of detected units across repeated cycles, such that a unit of time detected on one occasion is closer and closer to being identical to a unit of time detected on any other occasion. By synchronization, we refer to consistency of registered time across multiple clocks in different locations, such that the time registered by a clock in one airport (for example) will be the same as the time registered by a clock in another airport. The issue of synchronization is especially important in relation to the development and use of standardization in metrology. An example of standardization that is familiar to most readers is the establishment of time zones. For a general discussion of the history of standardization of clock time, see Levine (1997). Detailed discussion of the role of timekeeping standardization in physics, and of the technical aspects involved in harmonizing metrology with contemporary physics can be found in Ashby (2003), or Audoin and Guinot (2001, Chapter 3). All standards for measurement are, by their nature, conventional and, therefore, are extrinsic to the phenomena being measured (Tal, 2016).

### 2.1 Earth rotation

The rotation of the Earth causes the sun to move across the sky from morning to evening. Features of the layout that protrude from the ground will cast shadows on the ground, and the rotation of the Earth will cause those shadows to move. A sundial takes advantage of this natural effect to provide an indication of solar time. Generally, a sundial is an object that has been created to cast a clear shadow on a flat surface, and is oriented in such a way that the solar shadow moves at a constant speed during the day. The surface on which the shadow falls is marked with a “clock face” consisting of equally spaced lines, such that counting the lines that the shadow has crossed (i.e., those behind the current position of the leading edge of the shadow) yields the number of hours that have passed since the sun rose. One obvious limitation of the sundial is that it works only when the sky is clear. Another limitation is that sundials are not useful for indicating smaller units of time, such as minutes or seconds.

Sundials exhibit crude precision (i.e., sharpness of the shadow), and low accuracy (e.g., hours vary across the seasons). Their synchronization can be high (as for sundials located along a given line of longitude) or low (as for sundials located along a give line of latitude).

### 2.2 Physical flow

The limitations of sundials can be overcome using clocks that are not tied to planetary rotation but, rather, to physical flow that occurs at regular rates. The best-known examples are the flow of water and the flow of sand. Water clocks have existed at least since Classical Antiquity. The creation of clocks based on the flow of sand (i.e., the hourglass) emerged along with the ability to manufacture suitable glass vessels. Water clocks generally were graduated, that is, inscribed with a series of regularly spaced markings similar to those on a sundial (e.g., McNown, 1976). Hourglasses also could be graduated. Both types of clocks tended to be delicate, often messy, and were subject to mechanical perturbations (e.g., bumping). In addition, both types of clocks required frequent human intervention, to refill the water vessel or invert the hourglass. Finally, both are impractical for measuring long periods of time, such as days, or short periods of time, such as seconds.

Water clocks and hourglasses are characterized by crude precision, low accuracy, and poor synchronization.

### 2.3 Mechanical oscillation

Sundials, water clocks, and hourglasses register time using unidirectional movement: the daily progression of the sun from east to west, and the flow of water or sand under the influence of gravity. A major change in the nature of timekeeping occurred with the invention of clocks that registered time in terms of mechanical oscillations. In such clocks, bi-directional oscillations are transformed into unidirectional rotation through some type of escapement, such as a verge and foliot, or an anchor escapement (Cipolla, 2004). Oscillations are powered by a weighted pendulum, or the tension stored in a spring.

Through refinement across many centuries, mechanical clocks provided advances in precision, accuracy, and synchronization, eventually yielding synchronization of clocks across great distances, which enabled dramatic improvements in marine navigation (e.g., Taylor, 1962) and terrestrial scheduling (Levine, 1997).

### 2.4 Electrical oscillation

The 19th century saw the development of pendulum clocks that were powered by electricity. In the 20th century, the pendulum was eliminated. Rather than registering time through electrically-powered mechanical oscillations, in the synchronous electric clock time is registered in terms of the 60 Hz cycle of alternating current. In contemporary life, the overwhelming majority of electric clocks are of the synchronous electric variety.

Electrical clocks provided improved accuracy, and enabled a new type of synchronization, in which slave clocks were driven by a master clock, as in schools, factories, or office buildings (e.g., Ishibashi, 2014; Macii et al., 2009).

### 2.5 Crystal oscillation

Synchronous electric clocks were largely replaced as precision timekeepers with the development of solid-state clocks in which time was registered in terms of oscillations of quartz crystals. Crystal clocks provided improved precision, accuracy, and synchronization.

### 2.6 Atomic oscillation

The current standard for timekeeping is the atomic clock, also known as the Cesium clock. Rather than being based on the oscillations of macroscopic objects (e.g., pendula) or crystals (e.g., quartz), the atomic clock, as its name suggests, is based on the oscillation of atoms, specifically, on changes in state of atoms of Cesium-133 when excited by a beam of microwave radiation. Cesium clocks provide levels of precision and accuracy that enable the synchronization of processes that are built around extremely small units of time, such as navigational and telecommunications satellites.

Our review reveals that timekeeping technology consists in the detection of Events_P_, from the rotation of the Earth to the oscillation of Cesium atoms. Table 1 summarizes the relevant facts. Timekeeping technology has changed dramatically over the centuries, but in one respect it has remained unchanged: Clocks rely on detection of Events_P_. Clocks are not sensitive to the hypothetical passage of time that is extrinsic to Events_P_. In the Principia Mathematica Newton claimed, “absolute, true, and mathematical time, of itself and from its own nature, flows equably without relation to anything external,” [quoted in Gibson (1975), p. 299]. In modern metrology “a measurement of time … can only ever be accessible to us through the measurement of another quantity” (Audoin and Guinot, 2001, p. 7). We conclude that clocks detect things (Events_P_) that are claimed to happen “in” time but they do not detect the extrinsic frame within which Events_P_ are claimed to happen. Our discussion does not prove that clocks do not or cannot detect abstract, extrinsic time. It is always difficult to prove a negative. Rather, our discussion shows that all existing timekeeping technologies rely on the detection of Events_P_, and that the detection of Events_P_ is sufficient to support the functional utility of clocks in the measurement of time.

**TABLE 1:** Clock technologies through history, from earliest to most recent.

Timekeeping technology	Physical process registered	Type of event regularity	Mode of quantization	Role of convention
Sundial	Shadow movement	Earth rotation relative to sun	Shadow position on marked scale	Use, number and spacing of scale marks
Water clock	Flow of water	Constant flow rate	Water level relative to marked scale on vessel wall	Number and spacing of scale marks
Hourglass	Flow of sand	Constant flow rate	Moment of sand depletion	Volume of sand, flow rate (size of orifice)
Pendulum clock	Mechanical oscillation	Stable oscillation frequency	Escapement transforms pendulum motion into discrete “ticks”	Choice of pendulum frequency (length), number of ticks per s
Spring clock	Mechanical tension	Spring tension	Escapement transforms spring tension into discrete “ticks”	Number of ticks per s
Electric clock	Alternating electrical current	60 Hz current oscillation	Counting of oscillation cycles	Choice of current oscillation frequency, number of cycles per s
Quartz clock	Crystal piezoelectric oscillation	Material oscillatory cycle	Counting of oscillation cycles	Choice of crystal cut and resonance frequency, number of cycles per s
Atomic/Cesium clock	Atomic resonance of Cesium-133	Transition of Cesium-133 atoms between energy states	Counting of cycles of microwave radiation related to Cesium-133 transitions	Conventional (SI) definition of *s* in relation to Cesium-133 transition frequency

Given that clocks are not sensitive to absolute, extrinsic time, then What is it that they detect? We submit that clocks detect events, but not the events of general relativity (i.e., Events_GR_). Rather, we claim that clocks detect Events_P_. Given that Events_P_ includes Events_E_, it may be that clocks bear theoretically important relations to Gibson’s arguments about ecological physics.

## 3 Time for perceiving and acting

Gibson claimed that living things readily perceive Events_E_, but that we cannot perceive the extrinsic time that is used in general relativity: “There is no such thing as the perception of time, but only the perception of events and locomotions,” (Gibson, 1975, p. 295). Similarly, “the flow of abstract, empty time, however useful this concept may be to the physicist, has no reality for an animal” (Gibson, 1979, p. 12). Gibson also argued against the concept of the instant, or the instantaneous, claiming that these concepts conformed to mathematical abstractions, and were not part of physical reality: “nothing is instantaneous” (Gibson, 1975, p. 299). In physical reality, Events_P_ (including Events_E_) are not instantaneous. The continuous, non-discrete passage of time can be observed without clocks, as when we attend to the rising or falling tide, or when we observe the movement of shadows cast by sunlight across interior walls or floors or, indeed, the movement of the sun and moon across the sky. Continuous time also can be observed in more rapid Events_P_ and Events_E_, such as the fall of rain or the flight of birds. Non-humans also are sensitive to these continuous manifestations of time. Non-humans are not sensitive to the extrinsic quantization of time that is displayed by clocks. Indeed, the same is true of children, who must be taught to understand clocks, or to “tell time.” But children, like non-humans, do not need to be taught to perceive and understand the passage of time in day/night cycles, in flowing water, or in impending collision (e.g., Schmuckler and Li, 1998).

Subjective experience is divided into past, present, and future, but patterns in ambient arrays have no such divisions (Gibson, 1966, 1975, 1979). Patterns in ambient arrays often are specific to things that have happened (e.g., patterns relating to animal tracks, or to brushstrokes), but also to things that have not yet happened (e.g., impending collision), while things that “are happening” typically cannot be dissected into any discrete “now” (e.g., ongoing dynamic patterns in ambient arrays arising from locomotion, or from dynamic occlusion, or from rhythmic movements). “The natural input to the perceptual organ is a changing array of stimulation–not a stationary one,” (Gibson, 1966, p. 146; cf. Lee, 1980). The same logic applies to the underlying physical ontology: The flow of the Events_P_ that structure ambient arrays does not comprise categorical distinctions between past, present, and future.

Of critical importance for our analysis is the fact that the perceiving and controlling of temporally constrained behaviors does not appear to depend upon the perceiving of extrinsic time. Lee (1976) demonstrated the existence of patterns in the optic array that were deterministically related to “time to contact,” that is, the time remaining before contact of some object or surface with the point of observation. Such physical events give rise to characteristic patterns of change in patterns in ambient arrays, yielding what are called τ variables (e.g., Lee, 1990). A widely understood feature of the τ variable in optics, as well as in acoustics (Stoffregen and Pittenger, 1995), and haptics (Cabe and Pittenger, 1992), or in the global array (Stoffregen et al., 2017) is that the temporal information that it provides is continuous rather than discrete: The information does not exist in instantaneous patterns in ambient arrays, but only in changes in patterns over time. Another important fact is that the τ function has no extrinsic units. Time-to-contact can be measured in discrete units (e.g., seconds) and those measurements can be of use to scientists, but such measurements are irrelevant to perceiving-acting in relation to impending collision (e.g., Schmuckler and Li, 1998; Stoffregen, 2003). Continuous control of impending collision has been shown to be consistent with the information that exists in τ variables across a strikingly wide range of situations, individuals, functions, and species. Examples include striking and catching balls in adults (e.g., Bootsma and van Wieringen, 1990; Oudejans et al., 1996; Peper et al., 1994), and in infants and toddlers (e.g., Ekberg et al., 2013; Kayed and Van der Meer, 2009; Savelsburgh and Van der Kamp, 1993), interpersonal actions, such as passing and tackling (e.g., Correia et al., 2011; Hendricks et al., 2012), walking (e.g., Montagne et al., 2004), driving (e.g., Fajen, 2005), and powered flight (e.g., Izzo and De Croon, 2012; Prowse et al., 2008), insects’ timing of landing maneuvers in flight (e.g., Goyal et al., 2023), and birds’ timing of impact with water (e.g., Lee and Reddish, 1981). In all those cases, the information about timing that is available in patterns in ambient arrays is sufficient to explain the levels of precision, accuracy, and synchronization that are observed in temporally-constrained actions, consistent with Gibson’s (1979) arguments about direct perception: that perception can be accurate without the addition of internal processing.

In non-humans, the regulation of behavior relative to time is not limited to situations involving time-to-contact. Birds migrate with the seasons, turtle eggs buried in the sand hatch at the full moon, diurnal species sleep at night and move around by day, sunflowers turn throughout the day to face the sun. None of those species are sensitive to time as it has been standardized by humans in clocks, and improvements in human horology and metrology yield no benefits for them. It might be argued that non-humans don’t need the levels of precision in timekeeping that humans require, but this argument is undermined by the data on non-human sensitivity to time-to-contact, as discussed above. More broadly, we can point out that Darwinian evolution has proceeded over geological time with many species surviving and reproducing without sensitivity to extrinsic time. If Natural Selection has produced species that are adaptive without being sensitive to extrinsic time, then perhaps extrinsic time is not an appropriate standard for the scientific study of perceiving and acting.

It sometimes is assumed that Events_E_ refer exclusively to inanimate motions that are external to a perceiver. That was not Gibson’s view. Gibson included the perceiver’s own actions in the category of Events_E_, including locomotion and manipulation, among many others. He also included the actions of other living things (e.g., Stoffregen et al., 1999), as well as interactions between a given perceiver and other living things (e.g., Shockley et al., 2003). The fact that Events_E_ are extensive in time is reflected in the fact that Events_E_ can exist at many different time scales, from the very short to the very long. Finally, Events_E_ at different time scales are nested (Gibson, 1979, p. 94). An example might be flying from New York to Paris. The event begins at lift-off and ends at touchdown. Yet within that multi-hour Event_E_ are nested many shorter Events_E_, such as climbing to cruising altitude, changing heading to avoid storms, the serving of meals, communications with Air Traffic Control, descending to the runway, and so on.

## 4 Time in information processing

Gibson’s approach to time and temporality differs from more traditional work in psychology and cognitive neuroscience that emerges from concepts of information processing. Within information processing approaches, time and the perceiving of time typically are considered to be cognitive functions that influence awareness, cognition, and decision making. Research proceeds from the assumption that living things attempt to obtain knowledge about the extrinsic time of general relativity. For example, Pendleton and Clayton (2026, p. 1) offered a broad review of “temporal perception, cognition, and memory.” Their review began with a clear statement: “Physical time is a fundamental dimension of the universe that is linear and measured objectively by atomic clocks and astronomical cycles” (p. 2). In their analysis, the experience of time by living things was “a fluid and subjective construction” (p. 2). The bulk of their review was focused on conceptual and empirical details relating to the hypothetical construction of subjective impressions of time. Like Gibson (1979), they asserted that “time cannot be directly observed” (Pendleton and Clayton, 2026, p. 6), but from this premise they focused exclusively on the presumed sociological, psychological, and neuropsychological construction of internal estimates of time. In a review of developmental research, McCormack and Hoerl (2017) argued that children develop internal representations of time, similar to the subjective construction of time advocated by Pendleton and Clayton (2026). McCormack and Hoerl argued that very young children understand time in relation to events but that at around 5 years of age they begin to exhibit “an event-independent understanding of time” (p. 297) that constitutes “mature temporal cognition” (p. 300).

Within this information processing tradition, empirical research has focused on subjective judgments of duration, that is, the subjective awareness of the passage of time, often with reference to a hypothetical internal clock (e.g., Gibbon et al., 1984). Many studies have manipulated factors that have been hypothesized to influence the subjective awareness of duration (e.g., Gerstner and Yin, 2010; Lejeune and Wearden, 2009). The study of timing in “motor actions” typically has been limited to reaction time and other behaviors that do not involve continuous timing of ongoing action (e.g., Buhusi and Meck, 2005), with a focus on how movement may influence the hypothetical perception of time, rather than on how temporal information might be used in the control of ongoing movement (e.g., Allingham et al., 2021; De Kock et al., 2021; Treisman et al., 1992). Many studies have attempted to identify so-called neural mechanisms relating to perceived duration, consistent with the idea that the nervous system includes a clock function (e.g., Buhusi and Meck, 2005). None of the authors cited in this or the preceding paragraph cited or discussed Gibson’s analysis of time.

Our review indicates that information processing-based approaches proceed from two assumptions. First, time is assumed to be abstract, extrinsic, and absolute. Second, psychological or subjective time is assumed to exist as the result of internal models, processes, and/or neural functions. The Ecological Approach to Perceiving and Acting questions both of those assumptions. Gibson (1975, p. 295) argued that the perceiving of extrinsic time is a “false question” that is “unanswerable” and “insoluble.” In addition, Gibson argued that time is intrinsic to Events_E_, such that time might be perceived directly, with no need for internal models, estimates, or dedicated neural functions. For Gibson, extrinsic time, as construed in general relativity, is explanatorily unnecessary for theories of behavior in animal-environment systems. Put simply, the extrinsic time of physics should not be treated as the default object of temporal perception. We do not suggest that clock time has no role in the behavioral sciences, but only that the behavioral sciences should not assume that clock time is the object of temporal perception.

## 5 Time and space

Our arguments about absolute, extrinsic time apply equally to absolute, extrinsic space, such as 3-dimensional Cartesian space, or polar coordinates. This point was made extensively by Gibson who, for example, argued against the classical idea that space is extrinsic to physical objects (Gibson, 1950, 1966, 1975, 1979). Accordingly, technologies used in the measurement of extrinsic space (e.g., meter sticks, tape measures, or laser interferometers) are subject to the same conceptual issues that we have discussed in relation to technologies used in the measurement of extrinsic time. Gibson also argued that extrinsic space does not structure ambient energy arrays and, therefore, cannot, in principle, be perceived directly. By contrast, as we have noted with respect to the temporal aspects of Events_E_, the spatial aspects of Events_E_ might be perceived directly.

It is important to point out, however, that Events_E_ always involve both temporality and spatiality (see, e.g., Gibson, 1979, p. 101). There are no events without time (i.e., nothing is instantaneous), and no events without spatial extent (Gibson, 1966). With respect to the possibility of direct perceiving, a common example concerns the speed at which a vehicle is traveling. Intrinsic speed is visible: Drivers can see whether they are going faster or slower than other cars, because relative speed structures patterns in ambient arrays. We don’t need (or use) a speedometer to tell us about how fast we are moving relative to traffic. But extrinsic speed is not visible: Drivers cannot see whether they are going faster or slower relative to arbitrary, extrinsic metrics, such as speed limits in miles or kilometers per hour. Metric speed is the product of distance and time (*v* = d/t). If, as we have argued, extrinsic time is not specified, then it follows that variables derived from extrinsic time cannot be specified. Metric speed has abundant utility in many human applications. The speedometer is a good example, as it is useful because it provides perceivable (i.e., visible) access to locomotion in relation to extrinsic metrics.

A related example concerns the literature on the perceiving of egocentric distance (e.g., Bingham and Pagano, 1998; Bingham and Stassen, 1994; Mantel et al., 2015). In this literature, researchers have identified patterns in ambient arrays that may be lawfully specific to “distance.” Importantly, information about distance is available only across observer movement. That is, specification is inherently spatiotemporal. Moreover, we would argue that the distance that is specified (and, as shown in the empirical studies, is perceived) is intrinsic. That is, participants in the experiments do not perceive, report, or act upon distance in extrinsic units, such as centimeters, or inches.

The same argument applies to so-called smart perceptual mechanisms, which (we claim) make possible the direct perceiving of intrinsic (but not extrinsic) aspects of Events_E_ (e.g., Bingham et al., 1989; Runeson, 1977; Zhu and Bingham, 2008). It follows that clocks are not analogous to smart perceptual mechanisms. As we have argued, clocks detect Events_P_. Accordingly, such devices might be considered to be “smart” detectors of Events_P_. But we do not use clocks to learn about (for example) the number of times a pendulum has swung, or the number of times an atom has oscillated. Rather, clocks are useful when we interpret those Events_P_ in terms of our concepts of absolute, extrinsic time. The fact that people choose to use the detection of Events_P_ as a proxy for extrinsic time is conventional. That is, the interpretation of the metrological detection of Events_P_ in terms of time is extrinsic to the actual activity of clocks, such that clocks might be interpreted as “smart” detectors of Events_P_ but cannot be interpreted as “smart” detectors of time.

## 6 Conclusion

In this article, we have sought to understand differences between general relativity and the ecological approach to perceiving and acting in relation to concepts of time. Discussions of complex scientific theories sometimes can be facilitated through the use of “plain English” thought experiments that can help to clarify some of the issues involved. Here, we consider thought experiments that can help to illustrate the deeper conceptual distinctions that (we claim) exist between general relativity and the ecological approach.

Presentations of general relativity often employ a thought experiment in which a passenger on a train experiences objects on the train as being stationary, while the same objects are experienced as being in motion by an outside observer who watches the train go by. General relativity is illustrated by pointing out that both observers are correct. The thought experiment is useful because it illustrates the claim that motion is not absolute, but can be defined only relative to some frame of reference. From Gibson’s (1966, 1975; 1979) perspective, that classic thought experiment has some limitations. Perhaps the most consequential is that the point of the story is the observers’ conscious awareness (or, by analogy, the measurement of motion by a device). Neither observer controls the motion of the train, and neither observer uses perceived motion (or stasis) in the control of any action. That is, in terms of behavior the motion of the train is extrinsic to both observers, who are passive observers only.

A different thought experiment is useful in illustrating Gibson’s claim that time and space are intrinsic to Events_E_. Consider a bat using echolocation to capture an insect in flight (e.g., Ghose et al., 2006). Both the bat and the insect are in motion relative to the Earth, and both the bat and the insect are in motion relative to the air mass. However, the bat and the insect also are in motion relative to each other, and it is this motion that governs the outcome of the encounter. In this thought experiment both observers also are actors. Both generate and control their own motion, and both seek to control the relative motion between them: The bat seeks to reduce their mutual distance, while the insect seeks to increase it. The concepts of intrinsic time and Events_E_ are illustrated by pointing out that the relative motion of the insect and the bat (including both spatial and temporal aspects) can be described (and, arguably, perceived and controlled) in terms that are intrinsic to the nested events that are involved, and without reference to any external, extrinsic frameworks, whether spatial or temporal.

We have argued that Gibson’s conception of time as being intrinsic to Events_E_ is not compatible with time as it is construed in general relativity. We have suggested that Gibson’s concept of Events_E_ can be understood as a subset of the larger category of physical events (Events_P_), which is itself distinct from events as they are understood in general relativity (Events_GR_). We claim that clocks detect Events_P_ and that they do not detect Events_GR_ or the absolute extrinsic time of general relativity. The idea that clocks detect only Events_P_ is consistent with Gibson’s (1975) claim that extrinsic time cannot be perceived by living things. We have reviewed extensive empirical evidence that is consistent with Gibson’s claim that the perceiving and controlling of timing in behavior can be accounted for without reference to any concept of time that is extrinsic to Events_E_. Finally, we have argued that Gibson’s concept of intrinsic time may be sufficient for understanding the perceiving and controlling of behavior by humans and non-humans, such that the extrinsic time of physics should not be treated as the default object of temporal perception.
